# Grain filling leads to backflow of surplus water from the maize grain to the cob and plant *via* the xylem

**DOI:** 10.3389/fpls.2022.1008896

**Published:** 2022-12-01

**Authors:** Gui-Ping Zhang, Mukti Marasini, Wei-Wei Li, Feng-Lu Zhang

**Affiliations:** Agricultural college, Hebei Agricultural University, Baoding, China

**Keywords:** driving force of grain dehydration, grain filling, maize grain, pedicel xylem, surplus water, flow back, grain dehydration mechanism, grain formation period

## Abstract

Rapid dehydration of maize grain is one of the main characteristics of cultivar selection for mechanical grain harvest; however, the dominant driving forces and mechanisms of grain dehydration before physiological maturity remain disputable and obscure. This study compared the grain moisture content and dehydration rate of coated treatment (no surface evaporation) and control grains. Meanwhile, the xylem-mobile dye was infused from stem and cob, and its movement was observed in cob, ear-stalk and stem xylem. The development dynamics of husk, grain and cob were analyzed to determine the mechanism of grain dehydration. The results showed that, from grain formation to 5-10 days before physiological maturity, the main driving force of grain dehydration of the early and middle-maturity maize cultivars was filling, followed by surface evaporation. In the dye movement experiment, the movement of the stem-infused xylem-mobile dye through the pedicel xylem was observed during but not after the grain formation period. Moreover, the cob-infused xylem-mobile dye moved to the ear- stalk and the stem *via* the xylem. There was a significantly positive correlation between grain filling rate and dehydration rate from grain formation to physiological maturity. According to these results, we proposed that in the grain dehydration phase driven by filling, the surplus water in the grain flows back to the cob *via* the pedicel xylem, of which some flowed back to the plant *via* the cob and ear- stalk xylem. This provides a new theoretical basis for selecting and breeding maize cultivars suitable for mechanical grain harvesting.

## Introduction

The mechanical harvesting system of maize (*Zea mays* L.) grain combines ear picking and threshing, which improves the production efficiency (Guo et al., 2017). This system represents the development direction of maize harvest (Ministry of Agriculture and Rural Affairs Information Office, 2021) and the key to understanding the mechanization changes in maize production. However, one of the main factors restricting the application of maize grain mechanical harvesting is the high breakage rate of grains (Chai et al., 2017), which is mainly affected by the grain moisture content at harvest (Wang and Li, 2017; Guo et al., 2022; Shi et al., 2022). For example, in China, the moisture content of maize grain at harvest is universally higher (≥ 30%), which is unsuitable for mechanical grain harvesting (Li, 2021b). Dehydration is one of the key processes of grain ripening and drying, determining the grain moisture content at harvest (Kang et al., 1986; Muchow, 1990). Several studies have explored the factors influencing grain dehydration, including meteorological factors, grain properties, and cultivation measures (Gao et al., 2018; Zhang et al., 2018; Zhao et al., 2018; Wang et al., 2020); however, a few have focused on the grain dehydration mechanisms. It has been argued that grain dehydration is related to grain filling (Kang et al., 1986; Cross, 1991; Li et al., 2014; Cao et al., 2019); however, some scholars holding the opposite view (Li et al., 2018; Wan et al., 2018; Li et al., 2021a). I.R. Brooking (1990) investigated the relationship between dry matter and moisture of grains and ears of 18 maize cultivars from silking to harvest, and divided the grain dehydration process into two stages. The results showed that the first stage of the water loss, representing the developmental dehydration process from grain filling to physiological maturity, was related to grain filling. The second stage was the physical process of grain dehydration, occurring after physiological maturity. Furthermore, a study by Cao et al. (2019) on the grain dehydration characteristics showed that the grain dehydration rate had a significant positive correlation with the grain filling rate (P< 0.01) from the initial rapid dehydration stage to the slow dehydration stage, but had no significant correlation with meteorological factors, indicating the physiological dehydration properties. However, at the later stages, the grain dehydration rate increased rapidly and was positively correlated with temperature-related meteorological elements and average sunshine hours but negatively correlated with rainfall, suggesting the net dehydration characteristics. Thus, the grain dehydration mechanisms have only been speculated through grain development properties and the correlation analysis between the dehydration rate, grain filling rate, and meteorological factors, which still require further evidence.

After maize floret pollination, the plant transports nutrients and water to developing grains through the pedicel vascular bundle. The pedicel exists at the caryopsis base and contains two outer phloem vascular bundles with a cluster distribution at their end (Zhang et al., 1999), which branches from the longitudinal vascular bundles of the cob near the grain side. Thus, pedicel is the direct supplier of grain nutrients (Shao et al., 2016). The water and nutrients are symplasmically unloaded from the phloem and transported to the parenchymal cells of the placenta-chalazal (P-C) region above the vascular bundle of pedicle through plasmodesmata. Thereafter, the water and nutrients are exported by the corresponding transporter into the apoplasmic space of the maternal–filial interface and retrieved by the corresponding transporter in the basal endosperm transfer layer (BETL), which transports them to the embryo and endosperm (Kiesselbach, 1980; Balconi et al., 2004; Ruan, 2022; Shen et al., 2022).

However, the water migration pathway in grains during the grain filling process is still controversial (Zhang et al., 2007). Some studies have reported that the water in the cereal caryopsis is mainly lost through evaporation from the caryopsis pericarp (Radley, 1976; Lee and Atkey, 1984), while other studies postulate that the water in caryopsis could be transferred to the maternal tissue (Meredith and Jenkins, 1975; Zhang et al., 2007). Furthermore, previous studies have suggested that physical damage or xylem blockage caused by growth pressure (growth strains) might reduce xylem transport (Lang and Düring, 1991; Coombe and Mccarthy, 2000; Drazeta et al., 2004; Knipfer et al., 2015). Also, the excess water in the storage sink could be exported through the phloem; however, separating the inflow of photosynthetic assimilates from the water outflow in the phloem could be difficult (Patrick et al., 2001). Crafts and Currier (1963) established the “pressure flow hypothesis,” which states that numerous assimilates are transported to the sink through the phloem with water as a solvent, and if the water consumption (growth and transpiration) of the sink is not very high that the water transported through the phloem can meet or even exceed the demand, the surplus water is presumed to flow back to the parent tissue through the xylem. However, the pathway that water passes through the xylem during this process remains obscure (Zhang et al., 2007; Finch-Savage, 2013). Earlier studies have found that the water flowing into fruits or other storage organs through xylem was limited or even eliminated (Wiersum, 1966; Ollat et al., 2002) during the rapid growth period with the fastest increase in volume. In grape berries, xylem sap was the main water source before veraison (discoloration, beginning of ripening, cell expansion after a short lag), while the phloem sap became the main water source after veraison (Keller et al., 2006). Thus, the decline of xylem inflow at the onset of ripening might be due to an increase in sink-driven phloem influx (Keller et al., 2015). Zhang and Keller (2017) revealed that a fraction of water transported into the berry through the phloem was used for berry growth and surface evaporation, and the surplus was recirculated *via* the xylem during grape berry development, consistent with the “pressure flow hypothesis.” However, further research is still needed to determine whether the migration pathway of water in cob and grain during maize grain filling and dehydration accords with the “pressure flow hypothesis.”

A grain-coated technology has been developed (patent number: 2018112795690) to demonstrate the previous inference about the driving force of grain dehydration. Since this technology is meant to prevent water loss from the grain pericarp, the grain moisture content and dehydration rate of the treatment and control were compared during the grain filling process. This demonstrated whether grain filling and surface evaporation are the main dehydration driving force before and after grain physiological maturity, respectively. Therefore, this study emulated the transportation of apoplast dyes (basic fuchsin solution) into the vines fruits (Keller et al., 2006; Keller et al., 2015; Zhang and Keller, 2017), by continuously injecting the xylem-mobile dye into stems (under ear- stalk) and cobs of maize plants. The movement of the dye in the cob, ear- stalk, and stem xylems was then observed. Moreover, the development dynamics of ear and grain were recorded, and the grain filling rate, dehydration rate, and the contents of soluble sugar and starch in grains were determined. The migration pathways of water in cob and grain were studied.

## Materials and methods

### Experimental design and plant materials

**Figure 1:**
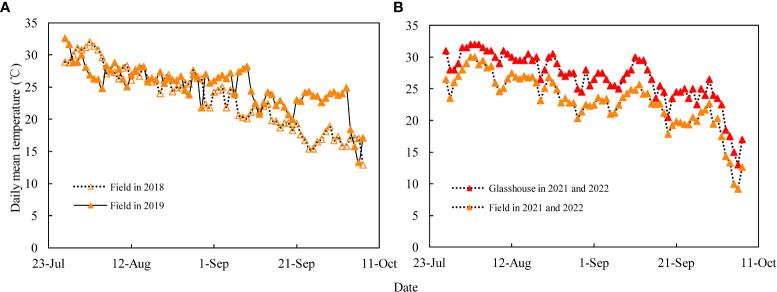
**(A)** Daily mean temperatures of the field in 2018 and 2019, and **(B)** averaged daily mean temperatures for 2021 and 2022 in the field and glasshouse during the grain filling stage.

The grain-coated experiment was conducted in 2018 and 2019 at the Qingyuan Experimental Station of Hebei Agricultural University, Hebei Province, China (38°49′N, 115°26′E; elevation 13m). The annual precipitation of the study year was 467.3 and 441.9mm, while that of the growing season was 387.5 and 344.8mm. The mid-maturing (JNK728) and early-maturing (XY779) maize cultivars were used as the experimental materials and were sown on 15 June. The planting area of each cultivar was about 200m^2^, with a row spacing of 0.6m and a planting density of 7.5×10^4^ plant hm^-2^. Controlled-release compound fertilizer, N-P_2_O_5_-K_2_O: 26-12-12, was applied at sowing as base fertilizer at the rate of about 750 kg hm^-2^. The no-tillage artificial sowing of maize was conducted using 2 seeds per hole, followed by irrigation to ensure seedling emergence, and singling at the 4-leaf period. The irrigation, weeding and pest control management during the maize growth period was the same as in the local production fields. The daily average temperatures of the test site (**Figure 1A**) were obtained from the small weather station (Watch Dog 2900 Weather Station) installed at the test site.

The dye movement experiment was conducted in 2021 and 2022 at the Innovation Experiment Park of Hebei Agricultural University, Hebei Province, China (38°49′N, 115°26′E; elevation 13m), using two maize cultivars, the mid-maturing (JNK728) and early-maturing (XY779). JNK728 was sowed on 30 May, while XY779 was sowed on 9 June 2021, and the two maize cultivars were sowed on 10 June 2022. The planting area of each cultivar was about 100 m^2^, and the cultivating practices were the same as those conducted for the grain-coated experiment, except for the irrigation. After first irrigation after sowing, the subsequent irrigation was performed when the soil moisture content decreased to 70% from the 100% field capacity. The temperature data of the glasshouse (**Figure 1B**) was recorded by a temperature recorder (Microlite USB DATA LOGGERS, LITE5032L), and that of the field was obtained from the small weather station (Watch Dog 2900 Weather Station).

### The grain-coated experiment

**Figure 2:**
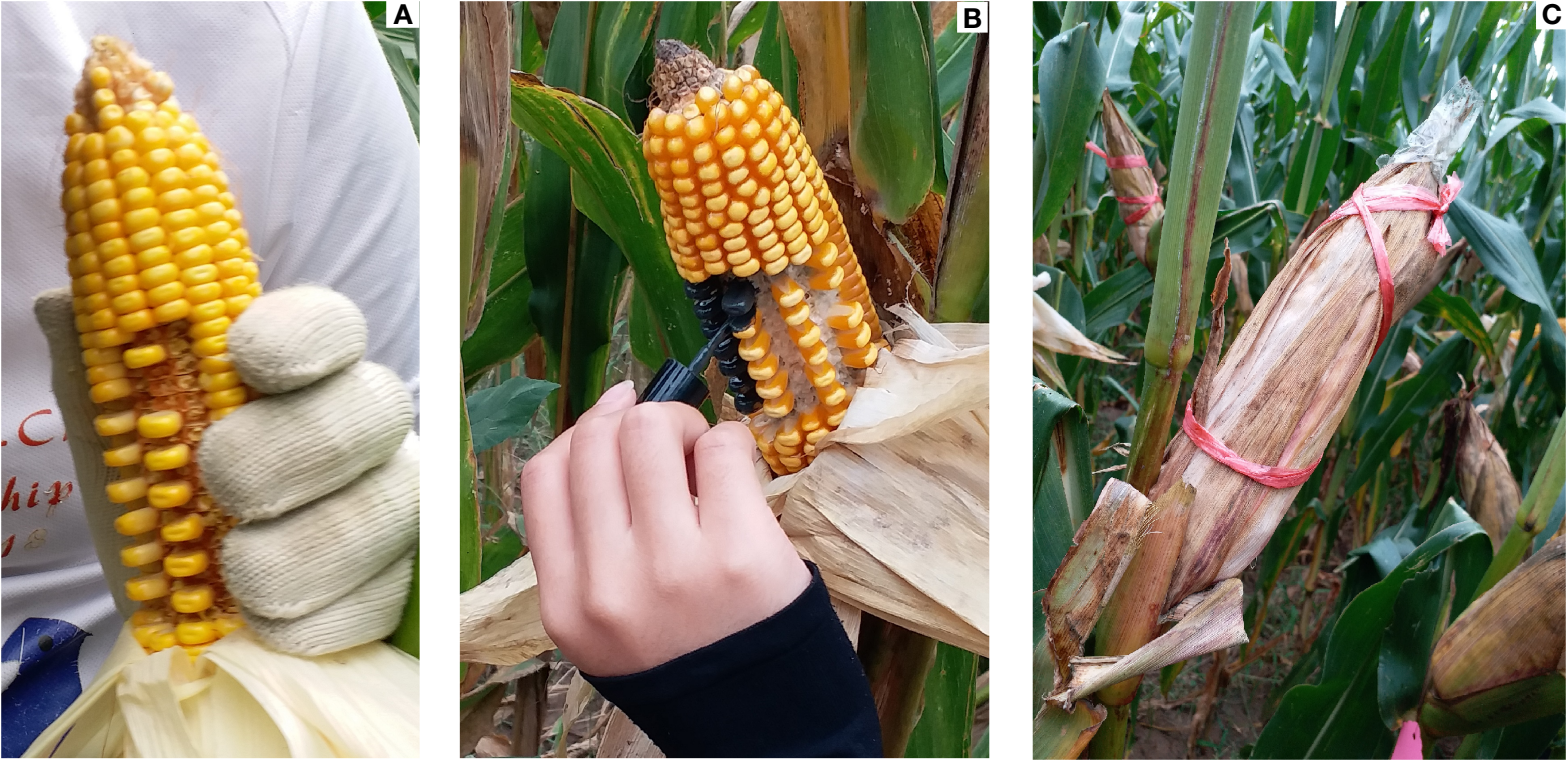
The grain-coated experiment process. **(A)** Alternated removal of middle grains in alternating rows, **(B)** coating of the 20 grains, **(C)** closed husk and bound the ear.

The plants with the same growth were marked in each plot of the two cultivars at the silking stage in the field. Starting from 35 days after pollination (DAP), ear husks of three marked plants selected from each cultivar plot were carefully stripped down layer by layer until the grain at the middle and upper ear position was exposed, with minimum destruction to the husk. The central ear portion was then used as the experimental treatment area. Tweezers were used to alternately remove the grains of the experimental treatment area in the alternating rows, to allow for the coated treatment of the remaining grains. We retained 40 individual kernels in the experimental area, of which 20 kernels were used for the coated treatment and the other 20 kernels were used as controls. After the grain removal operation, 20 remaining grains were completely coated by smearing evenly with a quick-drying liquid composite material (mainly including water, polyurethane - 1, sodium dodecyl benzene sulfonate, acrylates copolymer, sela ammonium chloride bentonite, phenoxyaethanolum) which did not affect the grain development. Upon drying, the material forms a resilient waterproof film that tightly wraps the grain, preventing the pericarp evaporation of the grain water. After the treatment, the husks were restored layer by layer and bound with string to maintain the original posture of the husk. Moreover, the husk at the ear top was capped with adhesive tape to prevent the grain mildew caused by the rain (**Figure 2**). The ears were treated for five days per five days until harvesting after grain physiological maturity and taken to the laboratory for further analysis. The waterproof coatings of the treated grains were removed, and 20 grains were collected from both the treatment and control for the fresh weight (FW) and volume determination. After that, the grains were oven-dried at a constant temperature of 85°C, and their dry weights (DW) were recorded. The grain filling rate (GFR), water content (WC), moisture content (MC), and dehydration rate (DR) were calculated based on the fresh and dry weight of the grains as follows (Li et al., 2018):


(1) $$\text{GFR(g }{}^{\circ}{\text{C}}^{-1})=\frac{{\text{W}}_{2}-{\text{W}}_{1}}{\text{A}{\text{T}}_{1-2}}$$

(2) WC(g)= FW-DW

(3) $$\text{MC(\%)=}\frac{\text{FW-DW}}{\text{FW}}x100\%$$

(4) $$\text{DR(\% }{}^{\circ}{\text{C}}^{-1})=\frac{\text{M}{\text{C}}_{1}-\text{M}{\text{C}}_{2}}{\text{A}{\text{T}}_{1-2}}$$

where, W_1_ and W_2_ are the dry weight of the preceding and the subsequent samples, respectively; AT_1-2_ is the accumulated temperature between the two samples; MC_1_ and MC_2_ are the moisture content of the preceding and the subsequent samples, respectively. The GFR, WC, MC and DR of the control were calculated according to equations (1), (2), (3), and (4). For the treated grain, the WC and MC were calculated according to equations (2) and (3), but the preceding dry weight (W_1_) and moisture content (MC_1_) in the equations (1) and (4) determining the GFR and DR used the control grain’s.

### The dye movement experiment

**Figure 3:**
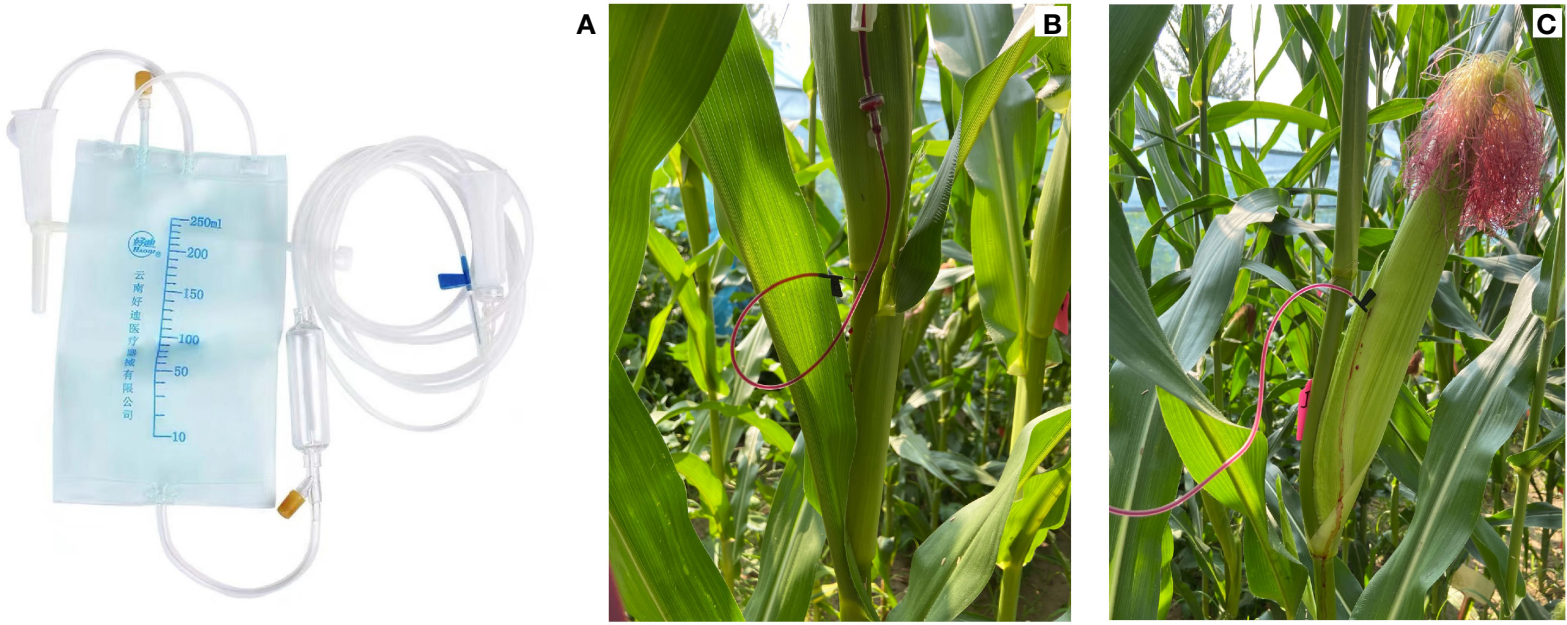
Dye movement experiments showing the infusion of the xylem-mobile dye *via* **(A)** the infusion apparatus into **(B)** the stem below the ear-stalk and **(C)** the ear cob.

The plants with the same growth were marked in each plot containing the two cultivars (JNK728, and XY779) during the silking period in the glasshouse at the Innovation Experiment Park. Nine labeled plants were selected from each plot starting from 5DAP, among which six plants from each plot were used for the dye-infused treatment, and the remaining three served as the control. Three of the six plants (from each plot) selected for the dye-infused treatment were continuously injected in the stem, 0.5 cm downward from the emerging internode of the ear-stalk, with 0.1% xylem-mobile dye (basic fuchsin solution, C20H19N3HCltermol.wt338Dqa) contained in an infusion bag. The other three were injected in the middle of the cob (**Figure 3**). The dye injected in stem and cob treatment lasted 3 days and 1 day, respectively. The infusion speed was adjusted to about 1drop per 15 seconds. After the infusion, the ears of the control and stem-infused treatment and the whole plant of cob-infused treatment were taken to the laboratory for analysis. The treatments were conducted per 5 days until harvested after physiological maturity. From 25 DAP, three representative plants were selected in the plot, and the withering state of the ear husk was continuously observed and photographed. Vernier caliper was used to measure the diameter of the middle part of the ear in its natural state (D_1_) and after tightening the husk (D_2_). The husk looseness (HL) was calculated as follows:


(5) $$\text{HL}=\frac{{\text{D}}_{1}}{{\text{D}}_{2}}$$

In the laboratory, the ears of the stem-infused plants were dissected longitudinally to observe the dye movement in the cob xylem. Conversely, the ears, ear- stalks and stems were dissected longitudinally for the cob-infused plants to observe the dye movement in their respective xylem. Subsequently, the ears of the three control plants were disintegrated to measure the fresh weights of husks, the middle 100 grains and the cob (the cob started from 15 DAP), and the volume of the 100 middle grains. The grain volume was determined by the drainage method, whereby an appropriate water volume was added to the measuring cylinder, and the new volume was determined after the grain was put into the cylinder. Thus, the grain volume was the difference between the two volumes. Each organ was then oven-dried to a constant weight at a constant temperature of 85°C, and their dry weights were recorded. The grain WC, MC, DR and GFR were calculated based on the dry and fresh weight of each organ, using the same method as that used in the grain-coated experiment. The dried seeds were crushed by an RS-FS1406 grinder, and then passed through a 100-mesh sieve, after which the contents of soluble sugar and starch were determined by anthrone colorimetry according to the national standard GB-5006-85 (Wang, 2021). Briefly, 0.05g of the dry powder sample of the grain was put into a small test tube, then 6~8 mL of distilled water was pipetted into the sample and incubated in boiling water for 30 min. Subsequently, the supernatant was obtained by centrifugation at 12,000×g for 10 min. The extraction was repeated once more, and the supernatant was diluted to 50 mL with distilled water, and used as soluble sugar sample for analysis. Thereafter, 8 mL of hydrochloric acid was added to the residue in the tube after soluble sugar extraction and was heated for 45 min in a boiling water bath. After cooling, the liquid and residue were transferred to the 50 mL bottle. After adding 8 mL of NaOH to the liquid and solid residue, the mixture was diluted with distilled water to 50 mL, and used as a starch sample for analysis. The sample to be tested was diluted with distilled water (1 to 6 times for soluble sugar and 2 to 8 times for starch) based on the number of days after pollination. After that 300 uL (soluble sugar) or 200 uL (starch) of diluted solution for analysis was pipetted out, and 800 uL of anthrone was added. After shaking, the samples were color-developed in a boiling water bath for 5 min (soluble sugar) or 6 min (starch) and cooled. The absorbance was then measured at 625 nm for soluble sugar or 620 nm for starch wavelength, whereby a mixture of distilled water and anthrone reagent was used as a blank control. The sucrose and glucose calibration curves were constructed for accurate quantification.

### Statistical analysis

Statistical analyses were performed using the Predictive Analytics Software (PASW) version 26.0 (IBM SPSS Statistics). The dry weight, volume, moisture content, filling rate, and dehydration rate of the grain between treatment and control plants were analyzed using the Paired-Samples T–Test for the grain-coated experiment. For the dye movement experiment, Pearson correlations analysis was performed to identify interrelationships among grain filling rate with grain dehydration rate; grain moisture content with husk moisture content, husk looseness; and grain dehydration rate with husk dehydration rate and husk loosing rate.

## Results

### Comparison of the grain moisture content and dehydration rate between the coated treatment and control

**Figure 4:**
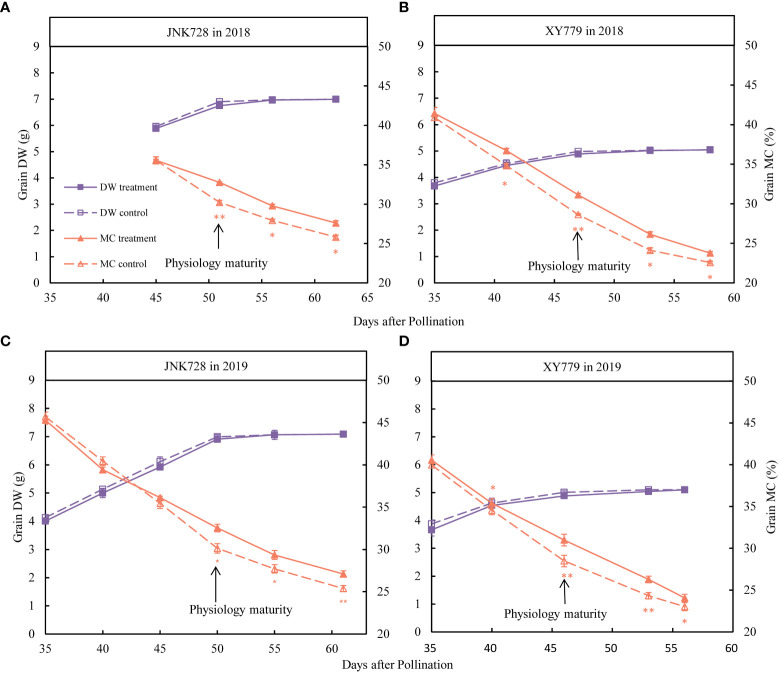
The Paired Samples T-test analysis showing the dry weight (DW) and moisture content (MC) of the 20 grains of the treatment and control collected from **(A, C)** JNK728 and **(B, D)** XY779, each, between the late stages of grain filling in 2018 and 2019. The single asterisk “*” indicates significance at 0.05, while the double asterisk “**” indicates significance at the 0.01 probability levels. The values are presented as means ± SE (n=3).

From the beginning of treatment (35 DAP or 45 DAP) to physiological maturity (**Figure 4**), the dry weight of the coated grains of JNK728 and XY779 cultivars was smaller than those of the control, but the difference was not significant, indicating that the coating treatment did not affect grain development. After physiological maturity (**Figure 4**), the grain dry weight of the treatment was similar to that of the control, however, the difference was insignificant. There was no difference in grain moisture content between the grain-coated middle maturing cultivar, JNK728, and the control before physiological maturity, but the difference became significant after the physiological maturity (**Figures 4A, C**). For the grain-coated early maturing cultivar, XY779, the grain moisture content was significantly higher than that of the control from 5 days before physiological maturity (**Figures 4B, D**).

**Figure 5:**
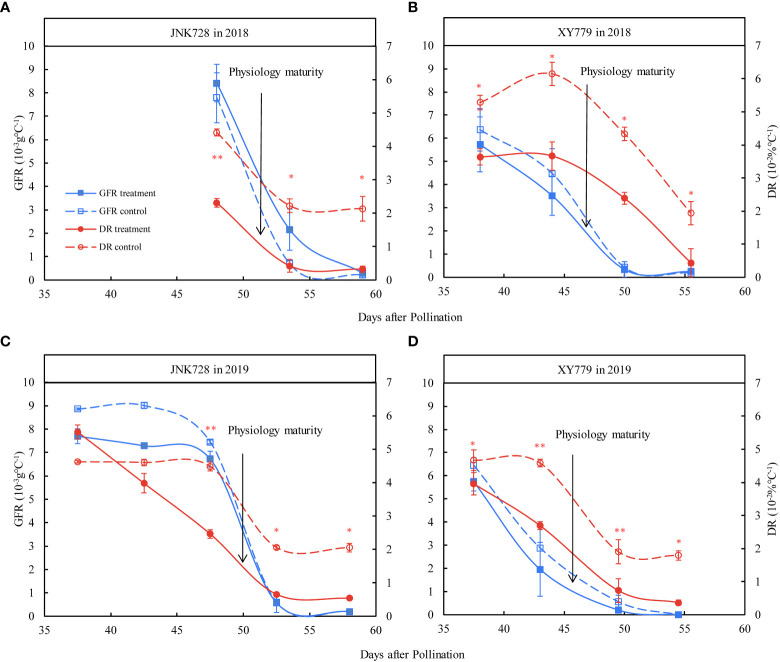
The Paired samples T-test analysis of the grain filling rates (GFR) and dehydration rates (DR) of the 20 grains of the treatment and control collected from **(A, C)** JNK728 and **(B, D)** XY779, each, at the late stages of grain filling in 2018 and 2019. The single asterisk “*” indicates significance at 0.05, while the double asterisk “**” indicates significance at the 0.01 probability levels. The values are presented as means ± SE (n=3).

Furthermore, the grain filling rate of the grain-coated JNK728 and XY779 was lower than that of the control, with no significant difference; however, JNK728 had a higher grain filling rate than that of XY779 (**Figure 5**). The grain dehydration rate of grain-coated JNK728 and XY779 was significantly lower than that of the control from 5 days (45 DAP) and 10 days (35 DAP) before physiological maturity to harvest, respectively (**Figure 5**). However, there was no significant difference in grain dehydration rate between the treatment and control of JNK728 and XY779 before 45 and 35 DAP, respectively.

### Movement of the dye in cob, ear-stalk, and stem

**Figure 6:**
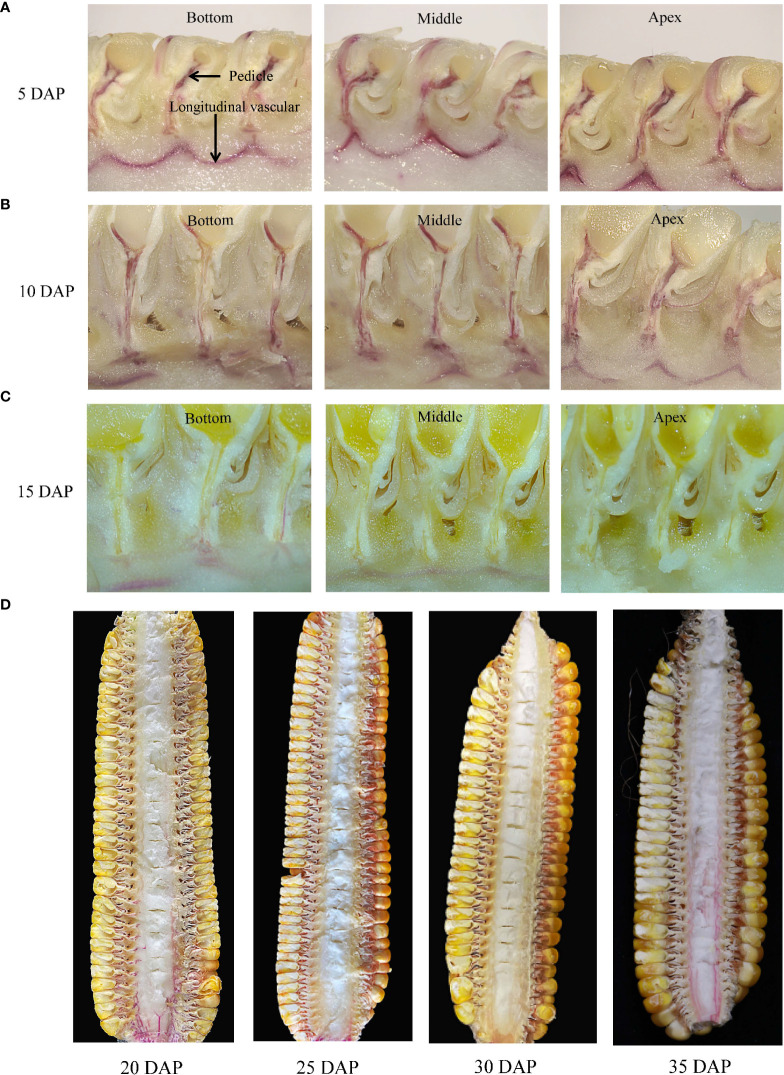
Movement of the xylem-mobile dye (basic fuchsin) infused through the stem below the ear- stalk. Longitudinal sections of different parts of the ears at **(A)** 5 days after pollination (DAP), **(B)**10 DAP, **(C)** 15 DAP, and **(D)** 20 DAP to 35 DAP for JNK728.

After the dye stem-infused treatment, we found that the dye could move through the pedicel xylem below the grain of JNK728 and XY779 within 10 days after pollination (**Figures 6A, B**). The dye was not observed in the pedicel, but was present in the longitudinal vascular bundles of cobs from 15 DAP (**Figure 6C**). In the early stage of maize grain development, i.e. the first 15 days after pollination, the embryo and endosperm rapidly develop into highly differentiated tissues from the initial zygote and endosperm nucleus. This stage is called the grain formation stage, and its duration varies among different maize cultivars (Rousseau et al., 2015; Li, 2018). After the emergence of the ear, the plant begins to transport water to the ear through the xylem. Water is transported to the grain through the pedicle xylem during but not after grain formation. At 25-30 DAP, the dye remained confined to the ear-stalk or lower parts of the cob (less entered the cob) but moved throughout the cob xylem after 35 DAP (**Figure 6D**). We found that water importation to the ear through the cob xylem ceased at 25-30 DAP, but recovered from 35 DAP.

**Figure 7:**
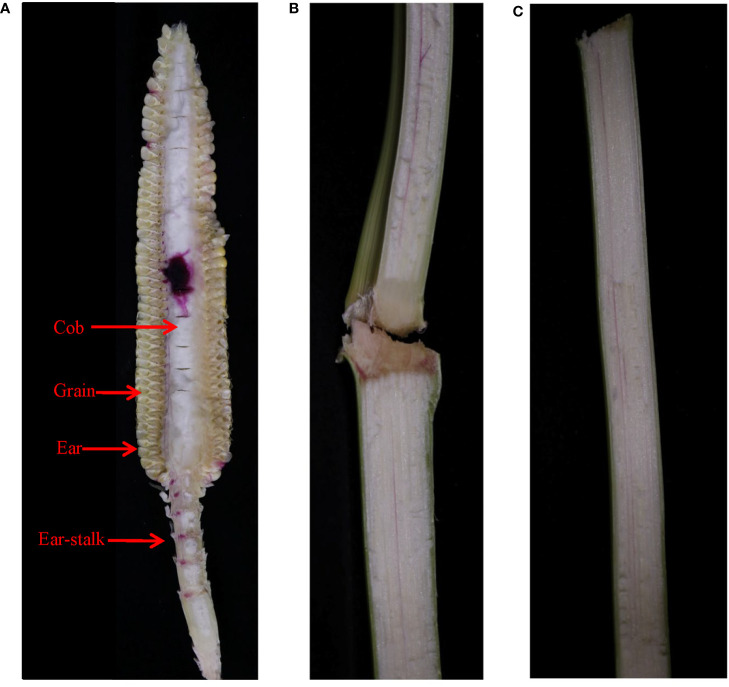
The movement of the xylem-mobile dye (basic fuchsin) infused through the cob of JNK728 through **(A)** the ear-stalk to **(B)** the stems bearing ear 1 day after the infusion, and further up **(C)** the stem 3 days after the infusion.

The dye infusion through the cob flowed back to the ear-stalk and stem bearing ear through the xylem (**Figures 7A, B**) from 5 DAP and could also flow back to the farther stem if the infusion time was extended to 3 days (**Figure 7C**). Thus, we inferred that there is both import and export of water in the ear *via* the xylem from pollination termination to 25 DAP and from 30 DAP to physiological maturity, with the net export being recorded at 25-30 DAP. Moreover, the surplus water flowed back to the plant from the ear through the xylem and was recycled by the plant during the grain development.

### Developmental features of each ear organ

**Figure 8:**
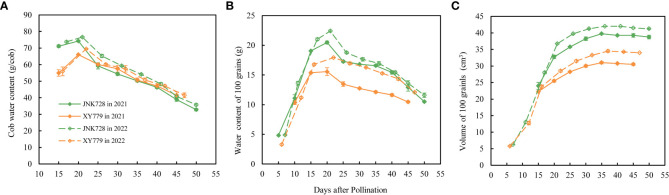
Water content of **(A)** the cob and **(B)** the 100 middle grains, and **(C)** the volume of 100 middle grains obtained from JNK728 and XY779 at the grain filling stage in 2021 and 2022. The values are presented as means ± SE (n=3).

The water content of the cob and the middle 100 grains of JNK728 and XY779 reached the maximum at 20 DAP (**Figures 8A, B**), while the volume of the middle 100 grains reached the maximum at 35 DAP (**Figure 8C**). This suggests that the ear needed less water for the subsequent developmental stages.

**Figure 9:**
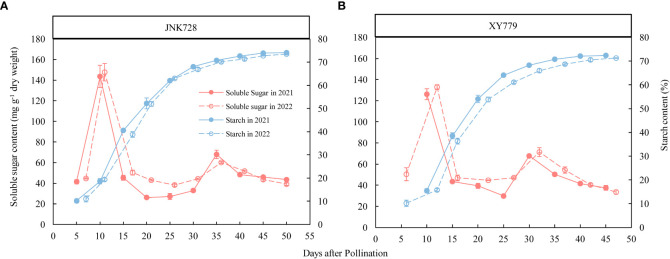
Soluble sugar and starch contents of **(A)** JNK728 and **(B)** XY779 grains at the grain filling stage in 2021 and 2022. The values are presented as means ± SE (n=3).

**Figure 10:**
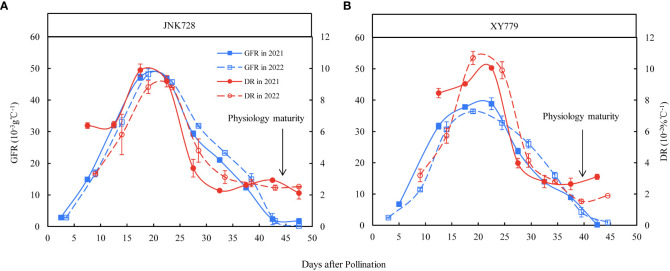
Grain filling rates (GFR) and dehydration rates (DR) of the 100 middle grains obtained from **(A)** JNK728 and **(B)** XY779 from grain formation to physiological maturity in 2021 and 2022. The values are presented as means ± SE (n=3).

The soluble sugar content of the two maize cultivars, JNK728 and XY779, showed a bimodal trend (**Figure 9A, B**) and reached their maximum at 10 DAP but decreased thereafter. The soluble sugar content increased gradually from 25DAP to its second peak at 35 and 30 DAP for JNK728 and XY779, respectively. After grain formation, the conversion rate of soluble sugar to starch in grain began to accelerate, and starch began to accumulate rapidly (**Figure 9**), inducing the onset of rapid grain filling (**Figure 10**). However, from 25DAP, the conversion rate began to decrease (**Figure 9**), and so did the filling rate (**Figure 10**).

**Figure 11:**
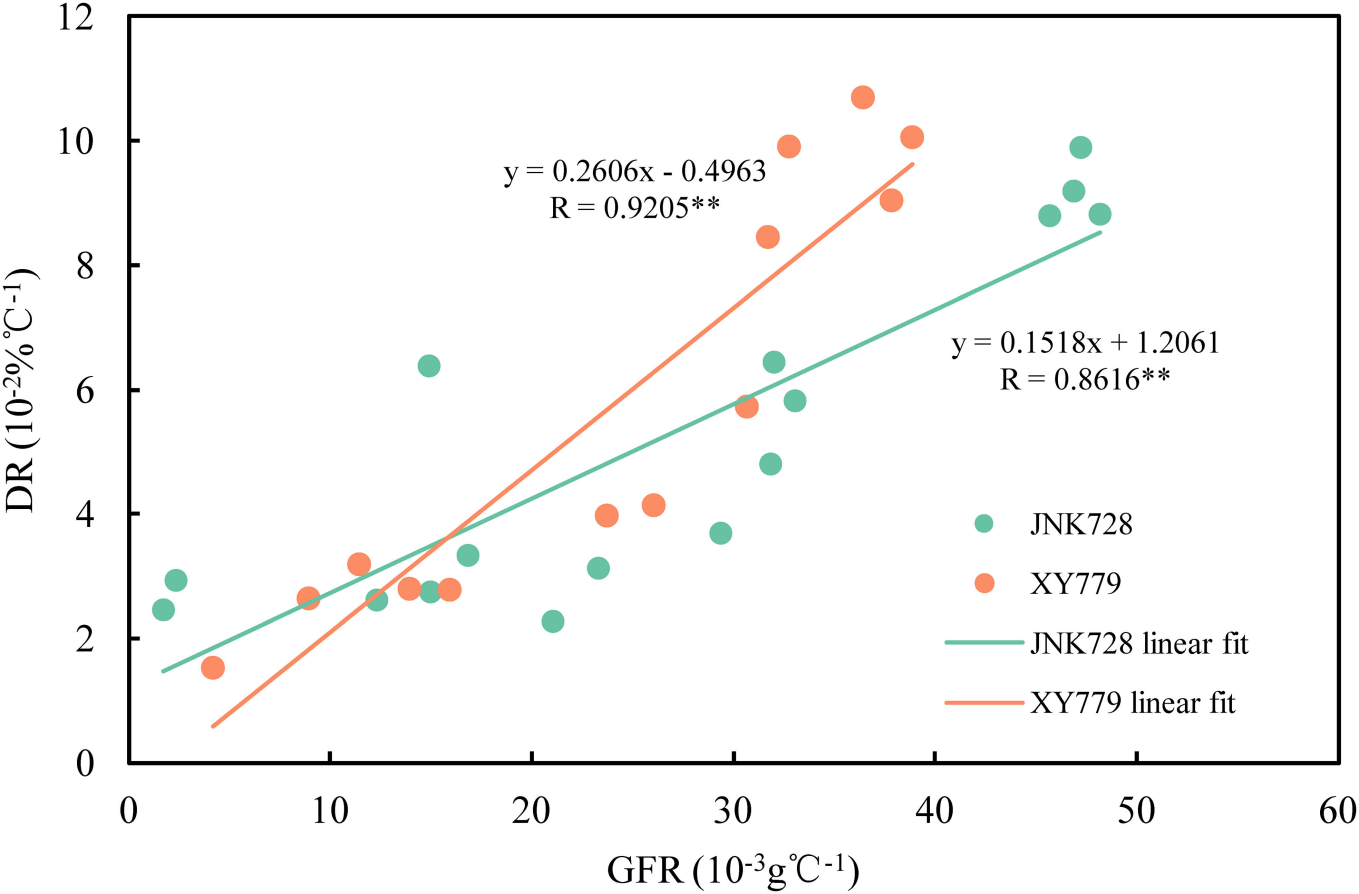
Relationship between grain filling rates (GFR) and dehydration rates (DR) of JNK728 and XY779 at the grain filling stage in 2021 and 2022. The double asterisk “**” indicates significance at the 0.01 probability level.

**Figure 12:**
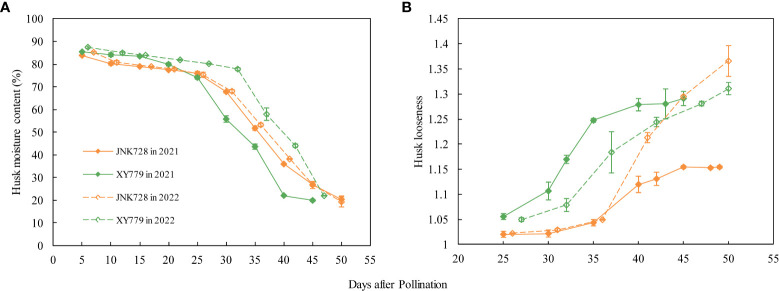
**(A)** Husk moisture content and **(B)** husk looseness of JNK728 and XY779 at grain filling stage in 2021 and 2022. The values are presented as means ± SE(*n*=3).

**Figure 13:**
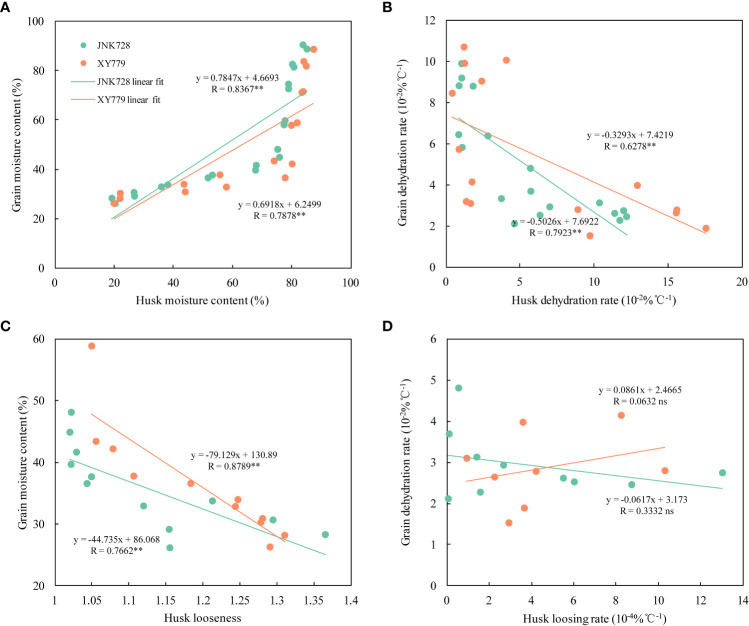
**(A)** Correlation analysis between the grain and husk moisture content, **(B)** the grain and husk dehydrating rate, **(C)** the grain moisture content and husk looseness, and **(D)** the grain dehydration rate and husk loosing rate of JNK728 and XY779 at the grain filling stage in 2021 and 2022. The double asterisks “**” indicate significance at the 0.01 probability level, while “ns” represents no significance.

The curve chart of the two maize cultivars, JNK728 and XY779, showed that the changing trend of GFR and DR were very close, with the maximum value at 15-25 DAP (**Figure 10**). A striking positive correlation was observed between the GFR and DR from the grain formation (after 10 DAP) to physiological maturity (**Figure 11**). From 30 DAP, the husk withering and drying process of XY779 was faster than that of JNK728; and, its moisture content was lower than that of JNK728. Additionally, XY779 had a higher loosing degree of the husk than JNK728 (**Figure 12**; **Supplementray Figure S1**) from 30 DAP. The husk of JNK728 and XY779 loosened rapidly at 35-40 DAP and 30-35 DAP, respectively. Correlation analysis between husk moisture and looseness and grain moisture traits in 2021 and 2022 showed a striking positive correlation between the husk and grain moisture contents (**Figure 13A**). However, the loosing degree of the husks exhibited a significant inverse correlation with the grain moisture content (**Figure 13C**). The correlation between the husk and grain dehydration rate was inversely significant (**Figure 13B**). There was no correlation between husk loosing and grain dehydration rates (**Figure 13D**).

The grain filling rates for JNK728 and XY779 were relatively high from grain formation to 35 and 30 DAP, respectively. The husk was not completely withered nor dried; thus, the ear was wrapped tightly by husk with high moisture content at this phase. Thus, the driving force of grain dehydration at this stage was grain filling, which was also demonstrated by the correlation analysis between grain filling and dehydration rate. After the grain filling and dehydration rates rapidly decreased, the husk moisture content decreased rapidly to less than 40% at 40 DAP, and the husk of JNK728 and XY779 loosened and withered completely at 40 and 35 DAP, respectively. Although the grain filling rate continued to decrease at this stage, the dehydration rate stabilized and started increasing with the progression of the developmental stages. Therefore, the grain filling rate decreases rapidly as the filling approaches the end, lowering its effect on grain dehydration. Along with the withering, loosing, and decrease in the moisture content of the husk, the main driving force of grain dehydration gradually changes from grain filling to surface evaporation. This finding shows that grain and ear developmental factors affect the main driving forces of grain dehydration (grain filling and surface evaporation). Consistent with the results of the grain-coated experiment, the main driving force of grain dehydration from grain formation to approximated physiological maturity (5-10 days before physiological maturity) is grain filling, which then changes to surface evaporation.

## Discussion

### The main driving force and pathway of grain dehydration

These results are consistent with the previous reports (Brooking, 1990; Cao et al., 2019), which stated that grain dehydration is divided into two stages, mainly driven by grain filling and surface evaporation, respectively. Before 5-10 days before physiological maturity, the grain filling rate, dehydration rate, and moisture content of early and middle mature maize cultivars were not significantly different from those of the control, a stage at which the dehydration process is driven dominantly by grain filling and the surplus water in the grain flows back to the cob through the pedicel. When the grain development approaches physiological maturity, the grain filling rate decreases to the lowest, resulting in striking differences in the dehydration rate and moisture content between the treatment and control plants. At this phase, grain dehydration is driven dominantly by surface evaporation.

From 35 DAP to physiological maturity, the grain filling rate of XY779 was lower than that of JNK728. Furthermore, the grain filling rate of XY779 at 10 days before physiological maturity was lower than that of JNK728 at 5 days before physiological maturity (**Figure 5**). In the later stage of grain development, the change of dominance from grain filling to surface evaporation in driving grain dehydration occurred earlier in XY779 than that in JNK728. As shown in **Figure 5**, the occurrence time of significant difference in grain dehydration rate between treatment and control of XY779 occurred five days earlier than that of JNK728. These findings are slightly different from those reported by Brooking (1990) and Finch-Savage (2013) because the demarcation point of different dehydration stages (physiological maturity) used in the present study was advanced by 5-10 days. Cao et al. (2019) studied the spring maize cultivar and showed that the light and thermal resources were sufficient to accelerate the dehydration rate in the late filling stage. However, the present study used summer maize cultivars; thus, there was no increase in the grain dehydration rate due to the lack of accumulated temperature in the late filling stage. Therefore, our results also differed from those reported by Cao.

### Water could be transported to the grain *via* the pedicel xylem only during grain formation stage

The basic fuchsin solution was continuously injected into JNK728 and XY779 through the stem below the ear- stalk and the cob. During grain development, the embryo and endosperm tissue are formed through cell division and tissue differentiation, and water inflow enhances their cell expansion, allowing for the accumulation of dry matter (Finch-Savage, 2013). Nutrients (assimilates) and water transported from the maternal plant to the P-C region through the pedicel vascular bundle are released to the extracellular space (apoplast) between the maternal and filial tissues, and then transported to the developing endosperm and embryo (Ruan, 2022; Shen et al., 2022). In the early stage of grain development, the phloem conductivity channeling of nutrients is low (Zhang et al., 2007); thus, the water supplied by the phloem is insufficient for grain development, prompting the xylem to provide water for the early expansion and growth of the grain (Westgate and Boyer, 1984). Therefore, in this study, the dye could enter the P-C zone following the water through the pedicel xylem during the grain formation period (**Figure 6A, B**).

High-concentration photoassimilates (sucrose and other assimilates) are loaded into the source phloem cells, creating osmotic potential gradients which attract water into the cells, thus increasing the turgor pressure in the source phloem. This results in a driving force, hydrostatic pressure gradient, which transports the photoassimilate solution to the sink organs (van Bel, 1996; Lalonde et al., 2003; Koch, 2004; Turgeon, 2010; Patrick, 2013). In sink tissue, sucrose unloading from the phloem decreases the solute concentration in the sieve tubes, resulting in water diffusion from the sieve tubes into the surrounding cells. This lowers the pressure and propels the bulk flow of solutes (sucrose and other assimilates) from the source to sink tissues (Lalonde et al., 2003; Patrick, 2013). At the grain formation stage (within 10 DAP), the grain content of soluble sugar increased rapidly (**Figure 9**). Due to the low grain filling rate, the starch accumulation was less, and the solute in the grain was mainly soluble sugar. This increased the osmotic pressure and lowered the water potential in the grain (Westgate and Boyer, 1986), enabling water supply through the pedicel xylem and phloem for the grain.

### The rapid conversion of sugar to starch in the grain after grain formation accelerated the phloem transport rate, preventing water supply for the grain through the pedicle xylem

The sugar was rapidly converted into starch, accelerating the grain filling rate after grain formation (**Figures 9**, **10**), consistent with the results of previous studies (Huo, 2016; Li et al., 2016). Patrick and Offler (1995) found that the sucrose absorption rate of seed apoplasts increased with the increase of polymer formation rate in pea cotyledons. The decrease in osmotic pressure of the apoplast sap stimulates the outflow of nutrients from the seed coat to the seed apoplast to match the rate of nutrient uptake by cotyledons (Patrick and Offler, 1995; Walker et al., 2000). The bulk flow of the sap from the phloem also supplies the water needed for volume growth in expansion sinks (Bret-Harte and Silk, 1994; Farrar et al., 1995; Pritchard et al., 2000). Studies have shown that most of the water supplied for grain development might be transported through the phloem (Pate et al., 1977; Jenner, 1982); showing that when the transformation rate of sugar to starch is accelerated, the osmotic pressure decreases in the grain, promoting the transport of sugar into the grain from the P-C zone *via* the apoplastic pathway. This decreases the osmotic potential of the P-C zone, thus accelerating the transport rate of the phloem. Then the water supply for the grain through the pedicel phloem is able to gradually meet the needs of grain development, and the pedicel xylem no longer transports water to the grain.

### At 25-30 DAP, the cob xylem ceases to transport water to the ear, possibly because the water import through the cob phloem could meet the water demand for ear development and evaporation

The grain filling rate of JNK728 and XY779 was high at 25-30 DAP because the former cultivar was in the later stages of rapid filling while the latter had just finished the rapid filling process (**Figure 10**). The grain volume of the two cultivars was close to the maximum value (**Figure 8C**), and their grain and cob water content reached the maximum at 20 DAP (**Figure 8A, B**). Moreover, their husks were not completely withered and loose and had a moisture content above 50% (**Figure 12**, **13**). We found that less water is needed for ear development and lost by evaporation, suggesting that the water imported through the cob phloem is sufficient or even exceeds the amount required for ear development at 25-30 DAP. Therefore, there was, almost exclusively, net water export from ear through the cob xylem, and the dye movement could only be observed in the ear- stalk or the lower parts of the cob. The soluble sugar content of grain began to increase at the later stages of the rapid filling phase at 25-30 DAP, reaching the second peak at 35 and 30 DAP for JNK728 and XY779, respectively. Thus, the transformation rate of sugar to starch began to decrease at 25 DAP, increasing the accumulation of soluble sugar in the grains (**Figure 9**). This also increases the content of soluble sugar in the P-C region, thus decreasing the phloem unloading rate (Patrick and Offler, 1995; Lalonde et al., 2003; Finch-Savage, 2013; Patrick, 2013). Lalonde et al. (2003) and Patrick (2013) reported that in the sink tissue, unloading sucrose from the phloem reduces the solute concentration in the sieve tubes, causing water diffusion from the sieve tubes to the surrounding cells, thus reducing the pressure in the phloem. When the unloading rate of phloem decreases, the osmotic pressure of the sieve tube decreases gradually, reducing or preventing water absorption through the cob xylem. Thus, there was only net water export through cob xylem from 25 to 30 DAP.

### The surplus water in grain and cob may flow back to the plant *via* the xylem

The dye movement experiment of the stem-infused plants showed that the cob xylem ceases and resumes transporting water into the ear periodically along with transporting the surplus water from ear back to the plant. The results prove that the cob xylem ceases importing water into the ear not because the xylem vessel is blocked but because the direction of water transport in the cob xylem changes from towards ear to towards plant. The cob may also stop transporting water to the grain through the pedicel xylem when the surplus water in grain is transported back to the cob *via* the pedicel xylem, but not because of the xylem vessel blockage.

The water loss from the expanded leaves to the atmosphere through transpiration generates negative pressure (Px) (Jupa et al., 2016; Keller et al., 2015) in the xylem *via* hydraulic pressure, which induces the surplus water in the sink tissue apoplast to flow back to the maternal plant through the xylem (Pate et al., 1985; Jenner and Jones, 1990; Keller et al., 2006; Zhang et al., 2006; Finch-Savage, 2013; Keller et al., 2015). Furthermore, the study by Zhang et al. (2007) suggests that the excess water in the sink tissue apoplast is transported to the phloem for recycling, which might involve the alternative opening and closing of the aquaporins corresponding to phloem unloading and xylem circulation. Therefore, we postulate that the osmotic pressure decreases during the grain filling due to the rapid transformation of sugar to starch. This causes the water in the grain to flow back to the cob xylem under the negative pressure produced by plant transpiration. Part of the water in the cob xylem is then transported to the phloem through the aquaporins and recycled, while the other part flows back to the maternal plant through the xylem to be recycled by the plant and the remaining is lost by evaporation. Thus, our findings support the “pressure flow hypothesis.”

The grain dehydration rate of JNK728 increased gradually from 35 DAP to 45 DAP, while that of XY779 remained stable initially and gradually increased thereafter from 30 DAP to 40 DAP (**Figure 10**). Studies by Brooking (1990); Feng et al. (2017), and Gao et al. (2018) showed that grain dehydration is related to meteorological factors, such as temperature and humidity. At the above stage, the husk moisture contents decreased gradually and the husks began to loose rapidly. The higher temperatures and lower humidity of the glasshouse promoted surface evaporation which accelerated grain dehydration. The glasshouse used for the dye movement experiment had higher temperatures than the field (**Figure 1**) where the grain-coated experiment was conducted. This shortened the growth period of maize in the glasshouse, leading to the early termination of the grain effective filling period and the onset of withering and loosing of the husks. Therefore, the conversion time of the main driving force of grain dehydration is based on the results of the grain-coated experiment.

## Conclusion

When photosynthates are transported to the grains through the pedicel phloem, the osmotic pressure of the grains increases, while the transport rate of the pedicel phloem is low, inducing the pedicel xylem to also transport water necessary for early expansion and development during the grain formation period (within about 10 DAP). After the grain formation stage terminates, the grain filling rate and the import rate of the pedicel phloem increase, and water import *via* the pedicel xylem gradually decreases and ceases at 15 DAP. The grain filling rate was accelerated so that the water supply for the grain through the pedicel phloem could meet or even exceed the needs of grain development. Meanwhile the transformation rate of sugar to starch was also accelerated, decreasing the osmotic pressure in the grains and inducing the water backflow from the grains to cobs through the pedicel xylem. Some of the backflow water in the cob is recycled by the phloem, some is lost through evaporation at the cob surface, while the rest flows back to the plant for reuse. There was a significantly positive correlation between grain filling rate and dehydration rate from the end of grain formation to physiological maturity. Combined with the results of the grain-coated experiment, these findings show that from grain formation to near the beginning of physiological maturity (5-10 days before physiological maturity), the main driving force of grain dehydration of the early and middle maturity maize cultivars is grain filling, which then changes to surface evaporation.

## Data availability statement

The original contributions presented in the study are included in the article/**Supplementary Material**. Further inquiries can be directed to the corresponding author.

## Author contributions

F-LZ: funding acquisition F-LZ: acquisition of the seed materials. F-LZ and G-PZ: designing the experiments. F-LZ, G-PZ, MM and W-WL: data collection. F-LZ and G-PZ: data analysis. G-PZ, F-LZ and MM: writing with contributions from all authors. All authors contributed to the article and approved the submitted version.

## Funding

This work was supported by the National Key Research and Development Program of China (2016YFD0300306).

## Acknowledgments

We would like to thank MogoEdit (https://www.mogoedit.com) for its English editing during the preparation of this manuscript.

## Conflict of interest

The authors declare that the research was conducted in the absence of any commercial or financial relationships that could be construed as a potential conflict of interest.

The reviewer JR declared a shared affiliation with the authors to the handling editor at the time of review

## Publisher’s note

All claims expressed in this article are solely those of the authors and do not necessarily represent those of their affiliated organizations, or those of the publisher, the editors and the reviewers. Any product that may be evaluated in this article, or claim that may be made by its manufacturer, is not guaranteed or endorsed by the publisher.
