# Supplementary figures and images for: Grain filling leads to backflow of surplus water from the maize grain to the cob and plant via the xylem
Source: Front Plant Sci. 2022 Dec 1;13:1008896. doi: 10.3389/fpls.2022.1008896 (PMC9762273; doi:10.3389/fpls.2022.1008896)

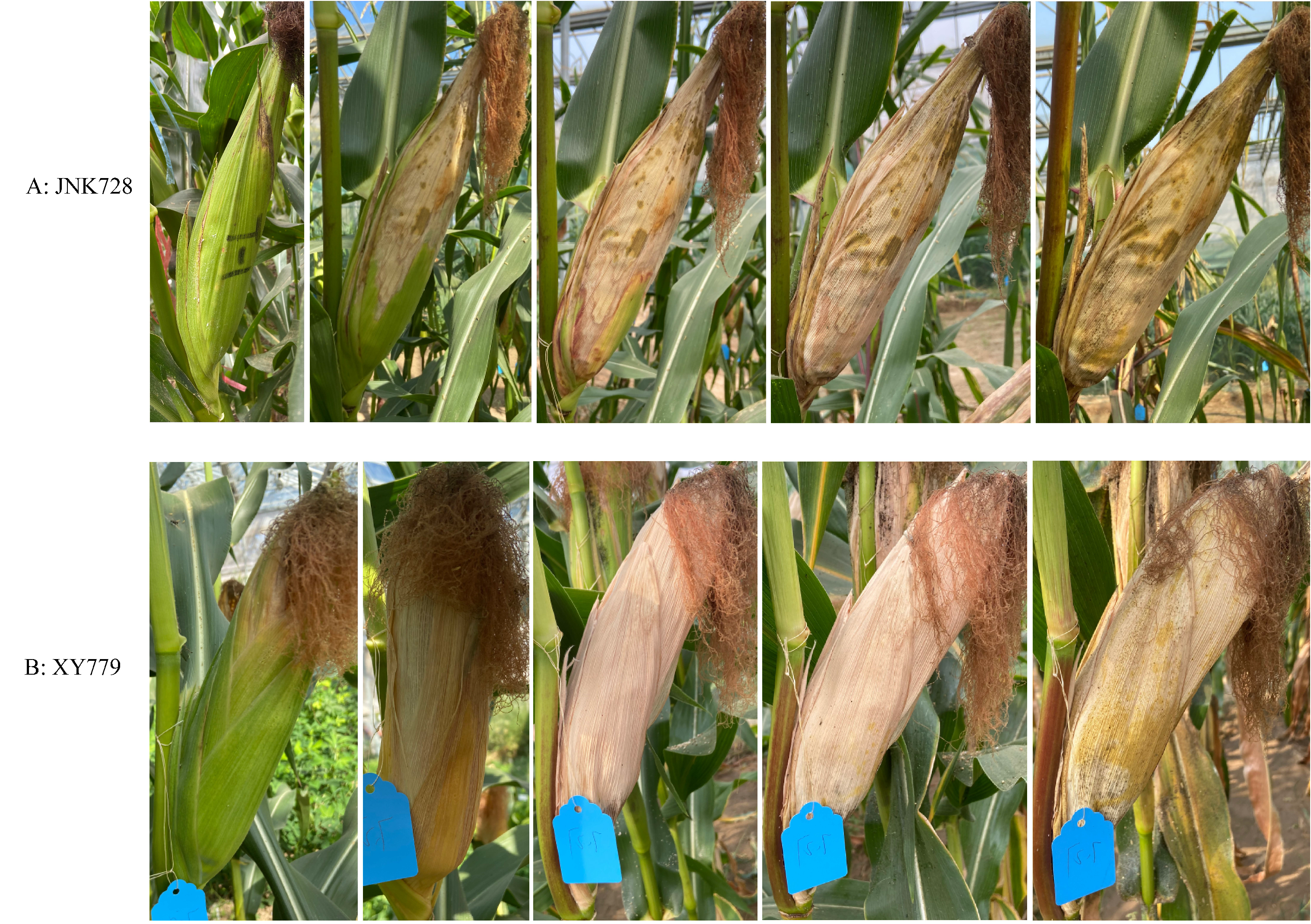

Supplement: Supplementary file 1 [file Image_1.tif]
